# Intravenous Magnesium Sulphate for Analgesia after Caesarean Section: A Systematic Review

**DOI:** 10.1155/2017/9186374

**Published:** 2017-12-03

**Authors:** Andrew McKeown, Vyacheslav Seppi, Raymond Hodgson

**Affiliations:** ^1^Rural Clinical School, University of New South Wales, Sydney, Australia; ^2^Port Macquarie Base Hospital, Port Macquarie, Australia

## Abstract

**Objective:**

To summarise the evidence for use of intravenous magnesium for analgesic effect in caesarean section patients.

**Background:**

Postcaesarean pain requires effective analgesia. Magnesium, an *N*-methyl-D-aspartate receptor antagonist and calcium-channel blocker, has previously been investigated for its analgesic properties.

**Methods:**

A systematic search was conducted of *PubMed*, *Scopus*, *MEDLINE*, *Cochrane Library*, and *Google Scholar* databases for randomised-control trials comparing intravenous magnesium to placebo with analgesic outcomes in caesarean patients.

**Results:**

Ten trials met inclusion criteria. Seven were qualitatively compared after exclusion of three for unclear bias risk. Four trials were conducted with general anaesthesia, while three utilised neuraxial anaesthesia. Five of seven trials resulted in decreased analgesic requirement postoperatively and four of seven resulted in lower serial visual analogue scale scores.

**Conclusions:**

Adjunct analgesic agents are utilised to improve analgesic outcomes and minimise opioid side effects. Preoperative intravenous magnesium may decrease total postcaesarean rescue analgesia consumption with few side effects; however, small sample size and heterogeneity of methodology in included trials restricts the ability to draw strong conclusions. Therefore, given the apparent safety and efficacy of magnesium, its role as an adjunct analgesic in caesarean section patients should be further investigated with the most current anaesthetic techniques.

## 1. Introduction

Magnesium, the fourth most common cation in the body, has been used in medicine and anaesthesia for a range of applications including seizure prevention in preeclampsia, tocolysis, asthma management, and dysrhythmias [1–3]. Magnesium sulphate also appears effective at improving postoperative analgesia [4]. The postoperative analgesic effect of magnesium appears to extend to nonobstetric patients undergoing general or neuraxial anaesthesia [1]. Magnesium has an antagonistic effect at the *N*-methyl-D-aspartate (NMDA) receptor [5], as well as calcium-channel blocker properties [6]. Antagonism at the NMDA receptor is thought to alter the mechanism of central hypersensivity and to subsequently decrease analgesic requirements including opioid consumption [7]. Postoperative shivering also appears to be decreased in patients given preoperative magnesium [8].

In patients who undergo caesarean section, adequate postoperative pain management allows early and sufficient ambulation and breastfeeding [9] and reduces complications including thromboembolic events, pneumonia, and poor bonding with the newborn while also improving patient satisfaction [9, 10]. Effective postoperative analgesia may enable patients to be discharged from hospital earlier following caesarean deliveries [11]. Consequently, several analgesia modalities and adjuncts, of which magnesium may be included, have been studied and reviewed for postcaesarean analgesia [9].

To our knowledge, no systematic review exists summarising the evidence for use of intravenous magnesium as an adjunct for postcaesarean section analgesia. Therefore, the aim of our review and our research question was to identify, evaluate, and summarise the evidence for the use of intravenous magnesium sulphate, compared to placebo control, for analgesic effect in parturients undergoing caesarean section and qualitatively review its effectiveness.

## 2. Methods

A systematic literature search on *PubMed*, *Scopus*, *MEDLINE*, *Cochrane Library*, and *Google Scholar* databases was performed using the search terms “(caesarean OR cesarean) AND magnesium”, all in title, with no language or other filters. All reference lists of articles found were also manually screened. The last search was conducted in May 2017. This review included only published studies and did not explicitly search for non-English trials, though no such trials were retrieved with the search strategy outlined.

Requirements for inclusion in this review were (1) intervention randomisation, (2) control group arm, (3) use of intravenous magnesium pre- or intraoperatively in parturients undergoing caesarean section, and (4) reporting on postoperative analgesia outcomes. Exclusion criteria included any trials that were retrospective or observational or studies on epidural or intrathecal use of magnesium sulphate.

All papers produced by the search methodology were screened. Papers not meeting the inclusion criteria were excluded. Each trial was assessed for risk of bias using the methodology described by the Cochrane Handbook [12] through examination of selection bias, performance bias, and detection bias. Postoperative pain and analgesic requirement outcomes, as reported in the original trials, were then compared qualitatively and are reported.

## 3. Results

**Figure 1:**
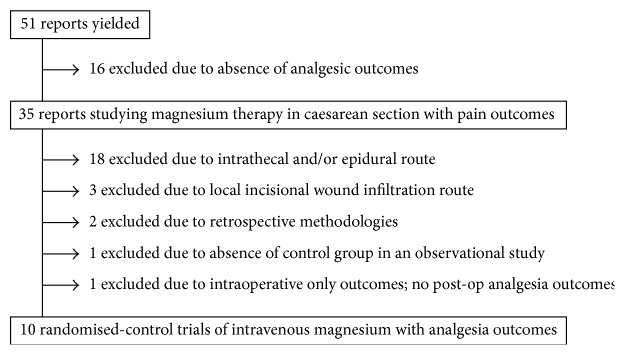
Report selection methodology.

Initial search methodology produced 51 studies which were then assessed for compliance with inclusion and exclusion criteria and were sorted as in Figure 1.

Twenty-one papers were excluded due to magnesium administration route other than intravenous. Sixteen were excluded due to absence of analgesic outcomes. One was excluded for reporting of intraoperative outcomes only with no postoperative analgesia outcomes reported [13]. Two were excluded due to retrospective or audit methodologies [14, 15]. Screening of references yielded one extra study which was subsequently excluded due to lack of placebo control in an observational study [16].

### 3.1. Trials Meeting Inclusion Criteria

Ten randomised trials meeting inclusion criteria were returned by the search methodology described. The assessment of risk of bias for each is outlined in Table 1. Seven of the ten trials were deemed to have low risk of bias and a high-quality rating per the GRADE approach [12]. Three trials, Agrawal et al. [17], Davoudi et al. [18], and Safavi et al. [19], were deemed to possess an unacceptably unclear risk of bias across all domains due to inability to discern whether the study protocol was insufficient or omitted. Subsequently, in line with the recommendations of the Cochrane Handbook for Systematic Reviews of Interventions, these studies were downgraded to a moderate quality rating per the GRADE approach [12] and excluded.

The seven subsequently included trials (Table 2) encompassed a total of 530 patients, 260 of whom received intravenous magnesium sulphate preoperatively. Four trials administered magnesium concurrently with general anaesthesia [20–23] (240 patients: 110 receiving magnesium sulphate) while the other three were with neuraxial anaesthesia [11, 24, 25] (290 patients: 150 receiving magnesium sulphate).

### 3.2. Magnesium Administration Protocols

All seven trials administered placebo control and intravenous magnesium interventions preoperatively as a bolus. However, three trials also administered an ongoing magnesium infusion for 24 hours postoperatively following the preoperative bolus [11, 24, 25]. The trial by Paech et al. [11] included two intervention arms (high dose and low dose), both with bolus-infusion regimens.

Dosage regimens used by each trial are outlined in Table 3. Doses ranged from 25 mg/kg to 50 mg/kg. One study used a standard dose of 6 grams as a bolus [24] which would likely be the highest dose-to-weight studied. 50 mg/kg was the most commonly studied bolus dose, with four trials assessing a group with this dose [11, 20–22]. Of those with postoperative infusion protocols, two groups received a standard 2 g/hr infusion [11, 24], one group received 1 g/hr [11] and one group received a weight-based infusion of 10 mg/kg/hr for 24 hours [25].

### 3.3. Analgesic Requirements

Five trials measured time to first analgesia requirement (Table 4). The effect of magnesium on prolonging time to first rescue analgesia varied in significance between trials. Three of the five found significant prolongation compared with control, *p* < 0.01 [20, 24, 25]. The remaining two found IV magnesium comparable with IV placebo [11, 23].

Total analgesic requirement in the immediate 24 hours post caesarean was measured in six of the seven trials. Intravenous magnesium resulted in significantly less analgesic consumption than placebo in four of the six, *p* < 0.01 [20–22, 25]. Likewise, Elgebaly et al. [24] did not measure total analgesic consumption, though did measure and reveal significantly lower frequency of analgesic intake in their magnesium group (2.5) compared to control (3.0; *p* < 0.01) [24] in the first 24 hours post caesarean, which may be a comparable outcome. The two trials which did not reach significance included limitations; Helmy et al. [23] had the smallest intervention sample size (*n* = 20) of any trial, and Paech et al. [11] was the only trial to provide patient-controlled epidural analgesia (PCEA) postoperatively. It is unclear if these discrepancies may have resulted in failure to reach significance.

### 3.4. Ranked Pain Outcomes

All included trials used a visual analogue scale (VAS) to measure patient-ranked pain scores (Table 5). However, time points and frequency of measurements varied widely from trial to trial. Elgebaly et al. [24] measured VAS scores only at the time of first analgesia requirement and did not reveal a significant difference between intervention and control groups. Similarly, Paech et al. [11] only measured a VAS score at one time point for patient analgesia satisfaction at 48 hours postoperatively which did not reveal benefit in the intervention group compared to control (*p*=0.449).

The remaining five trials serially measured VAS scores. Magnesium intervention resulted in significantly lower VAS scores at the 24-hour time point in two trials [22, 25] (*p* < 0.05) and consistently lower VAS scores at multiple time points in the same trials (*p* < 0.05).

However, Mireskanadari et al. [21] reported lower VAS scores in the intervention group at one, six, and 12 hours (*p* < 0.05), but not at 24 hours [21]. Similarly, Elrahman and Youssry [20] reported lower VAS scores at six and twelve hours in magnesium patients (*p* < 0.05), but not at two or at 24 hours. Two trials, Paech et al. [11] and Helmy et al. [23], measured VAS scores at comparable values to the placebo-control group at several time points. However, these intervention arms do show discrepancies as outlined in Section 3.3.

### 3.5. Comparison with Other Interventions

Two of the included trials compared intravenous magnesium sulphate with other active intervention arms. Helmy et al. [23] included a group which received a bolus of IV ketamine 0.3 mg/kg (*n* = 20) which was compared with an IV magnesium sulphate 30 mg/kg bolus (*n* = 20) group and IV normal saline control group (*n* = 20). In regard to postoperative pain and analgesia, the performance of magnesium was inferior to that of ketamine in prolonging the time to first analgesia requirement, lowering VAS scores (at two and six hours), and decreasing total 24-hour analgesic requirements. Ketamine produced significantly more favourable results compared to control while magnesium failed to do so [23].

Elgebaly et al. [24] compared the efficacy of an IV magnesium bolus and infusion as an adjunct to 0.5% bupivacaine spinal with a combination of spinal bupivacaine and an intrathecal bolus of 25 μg spinal fentanyl for postoperative analgesia. Magnesium performed comparably to fentanyl; both interventions prolonged time to first analgesic request and reduced total 24-hour analgesic requirements. Magnesium, however, produced fewer side effects than intrathecal fentanyl including pruritus and nausea and vomiting, while also causing less perioperative sedation in comparison to fentanyl (*p* < 0.01) [24].

### 3.6. Other Outcomes

In those trials measuring neonatal outcomes, magnesium performed comparably to placebo controls. Apgar scores were comparable in the five trials that reported them [11, 20, 21, 23, 25]. One trial routinely measured umbilical artery and vein pH, which was comparable between magnesium and control groups [11]. One trial routinely measured time to ambulate unassisted postoperatively, which found patients in the magnesium group ambulate sooner (4.2 hours) than control (6.3 hours, *p* < 0.01) [20].

### 3.7. Side Effects

Magnesium intervention did not result in toxicity in any of the trials. The incidence of postoperative nausea and vomiting or shivering was comparable to control in all trials that reported these outcomes. However, IV magnesium resulted in significantly greater surgeon-reported intraoperative blood loss than control in the trial by Paech et al. (500 mL median in high-dose magnesium group, 475 mL in low-dose group, and 400 mL in control group; *p* < 0.01) [11]. This increased loss is likely due to the tocolytic properties of magnesium sulphate [11]. This increased blood loss did not result in blood transfusion or additional uterotonic use in any case. Mireskandari et al. [21] and Elrahman and Youssry [20], however, recorded no significant difference in blood loss between patients receiving magnesium than control (*p* > 0.05). Maulik et al. [25] reported an increased incidence of intraoperative hypotension in magnesium intervention patients than control (*p* < 0.001), which was readily corrected with vasopressors [25].

## 4. Discussion

Magnesium, an NMDA receptor antagonist [5] and calcium channel blocker [6], has been utilised intravenously as an adjunct analgesic in various surgical procedures with varying results [26]. However, evidence for its analgesic role post caesarean section has not been systematically reviewed. This review consolidates the evidence for postcaesarean analgesia outcomes following intravenous magnesium which revealed conflicting results. The trend amongst available evidence suggests that analgesic requirements in the immediate 24-hour postoperative period may be reduced with IV magnesium. Beyond this, there is currently insufficient evidence for the role of preoperative intravenous magnesium for postcaesarean analgesia.

Unfortunately, a meta-analysis of the included studies was neither appropriate nor possible due to the small sample size of intervention groups and heterogeneity of methodology, inconsistent outcome points, and differing rescue analgesic practices and types. Assessment of publication bias, such as inclusion of a funnel plot, was not performed due to too few included studies. Subsequently, the aim of our review was to qualitatively assess the evidence for intravenous magnesium sulphate as a postoperative analgesic adjunct.

In regard to administration protocols, no argument can be made for using a bolus-infusion protocol over a bolus-only protocol on the evidence available. Likewise, neither anaesthetic type (spinal or general) revealed apparent benefit, although a study with direct comparison between these patient groups has not been conducted. A bolus of 50 mg/kg was the most commonly used dose; however, beyond citing similarity to dosing used for preeclampsia, no trials justified their dosing. As discussed by Helmy et al., a lower-dose bolus of 30 mg/kg may be insufficient [23]. The usual dosing of magnesium for preeclampsia is 4 grams as a loading dose and an infusion up to 24 hours at 1 g/hr [27].

Six of the seven included studies reported on total 24-hour postoperative analgesic requirement, and five of seven serially reported on postoperative VAS scores. With the exception of the study by Paech et al. [11] which was the only study to utilise PCEA and the study by Helmy et al. [23] in which an outcome difference may have been obscured by the low patient number in the intervention group, the remaining studies supported an analgesic effect in regard to improvement in 24-hour analgesic consumption and serial VAS scores. Our review found that there is insufficient evidence to support the role of intravenous magnesium in increasing time to first postoperative rescue analgesia requirement.

Other authors [22] have postulated that the superior analgesic efficacy of PCEA may have masked the analgesia effect of magnesium in the study by Paech et al. [11]. In addition, Helmy et al. [23] conceded in their study that failure to reach significant outcome differences from control may have been due to their low magnesium bolus dose (30 mg/kg compared to the most common 50 mg/kg) and lack of an ongoing infusion protocol [23].

The safety of IV magnesium for use as an analgesic adjunct can be translated from use elsewhere in obstetrics, or for analgesia in other surgical procedures, with reviews concluding no serious side effects at doses as high as 28 grams over 24 hours with no difference in morbidity compared with placebo [26, 28]. A Cochrane review of magnesium use in preeclamptic parturients supported its safety at doses up to 1 g/hr [27]. Further, magnesium for a range of indications has an established safety profile in parturients [3]. Increased surgeon-reported intraoperative blood loss was the single side effect identified in this review [11]. However, the effect of magnesium on uterine tone and subsequent requirement of additional oxytocin has been described elsewhere [29] and should be considered.

Strengths of our review include the exclusive criteria of intravenous magnesium for analgesic effect in parturients postcaesarean section. All trials were randomised and blinded, though not without some remaining risk of bias due to limited specifics on allocation concealment. The majority of trials reported on VAS scores, time to first analgesic request, and total 24-hour analgesic consumption allowing comparison.

There were several limitations in this review. Firstly, the majority of trials had small intervention groups (*n* = 20–42) perhaps limiting the ability to reach significance, for example, in the smallest group by Helmy et al. [23], or also introducing risk of benefit detection by chance [30]. The possibility that potential side effects may similarly have been undetected secondary to small sample sizes should also be considered. Secondly, there was inconsistency of end points and their reporting as well as differing modes of rescue analgesia. Further, there was variation in the dose and administration protocols used. There was also infrequent measurement and reporting of side effects including haemodynamics, nausea, vomiting, sedation, and shivering. Finally, the low control group pain scores in some trials [11] secondary to highly effective analgesia (PCEA) may also have confounded the ability to identify intervention benefit, given low baseline pain [31]—90% median pain control satisfaction in control group [11]. Specific measurements such as length of hospital stay and long-term (>48 hours) analgesia requirements failed to be reported in any trial with the exception of six-week follow-up data by Paech et al. [11].

The most significant limitation of our review to provide relevance to current practice is the failure of any study to utilise a modern approach to obstetric anaesthesia and postoperative pain management. Only three of the seven trials [11, 24, 25] were conducted with spinal anaesthesia, the current standard in modern obstetric anaesthesia [32], and only one trial [11] used intrathecal opioids (fentanyl) with their neuraxial technique. Longer-acting intrathecal opioids such as spinal morphine combined with local anaesthetic would be the most current and relevant mode of anaesthesia for testing IV magnesium as an analgesic adjunct [32, 33]. Likewise, no trial adopted a postoperative analgesia regimen in line with the current standard; a multimodal regimen including regular paracetamol and nonsteroidal anti-inflammatory drugs (NSAIDs) with rescue opioids [34]. It is difficult to comment whether the modes of anaesthesia utilised in the included trials impacted on their ability to identify intervention benefit; however, it undoubtedly limits the applicability of the results to current practice.

Future research agenda following our review and its limitations could comprise a randomised-control trial of intravenous magnesium as an adjunct to spinal anaesthesia using local anaesthetic and long-acting opioid followed by a multimodal analgesia regimen. Such a trial would ideally include two intervention arms: one employing a bolus-only regimen and the other employing a bolus-infusion regimen. A scheduled protocol to monitor for adverse effects is essential. Further, there remains a paucity of data on long-term outcomes including length of hospital stay and wound pain at six weeks which should also be addressed.

On balance, we conclude that preoperative intravenous magnesium has the potential to improve postcaesarean outcomes by decreasing total postoperative analgesic requirements. In the case of opioid rescue analgesia, this has the potential to decrease adverse effects including nausea, vomiting, sedation, and constipation while maintaining satisfactory analgesia [35]. However, this potential benefit needs to be further evaluated in forthcoming trials with adequate sample size and more current anaesthetic techniques. By ensuring satisfactory postcaesarean analgesia, patients may be able to ambulate and breastfeed earlier with greater patient satisfaction [9].

## 5. Conclusion

Preoperative intravenous magnesium sulphate may decrease 24-hour rescue analgesia requirements in patients following caesarean section with no serious adverse effects. Further trials utilising a current anaesthetic approach are required.

## Figures and Tables

**Table 1:** Assessment of risk of bias in selected studies based on Cochrane methodology and representation [12].

	Agrawal et al. (2015)	Davoudi et al. (2013)	Elebaly et al. (2011)	Elrahman and Youssry (2017)	Helmy et al. (2015)	Maulik et al. (2015)	Mireskandari et al. (2015)	Paech et al. (2006)	Rezae et al. (2014)	Safavi et al. (2017)
*Selection bias*										
Random sequence generation	?	?	+	+	+	+	+	+	?	?
Allocation concealment	?	?	+	+	+	+	?	+	+	?

*Performance bias*										
Blinding of participants and personnel	?	?	+	+	?	+	+	+	+	?

*Detection bias*										
Blinding of outcome assessment	?	?	+	+	?	+	+	+	?	?

*Attrition bias*										
Incomplete outcome data	?	?	+	+	?	+	+	+	?	?

*GRADE quality rating* [12]	M	M	H	H	H	H	H	H	H	M

+ = low risk of bias, ? = unclear risk of bias, – = high risk of bias, M = moderate, H = high.

**Table 2:** Included trials, participants, interventions, and outcomes.

	Total (*n*)	Anaesthetic type	Intervention groups (*n*)	Outcomes
Elgebaly et al., 2011, Egypt	90	*Spinal*	(i) IV magnesium sulphate bolus and 24 hr infusion (30)	(i) Total 24 hr analgesia use (paracetamol)
		(i) Bupivacaine 0.5% (2 mL)	(ii) 25 µg in 1 mL intrathecal fentanyl plus + NS IV (30)	(ii) Time to first analgesia
			(iii) Normal saline control (30)	(iii) Pain (VAS) score

Helmy et al., 2015, Egypt	60	*General*	(i) IV magnesium sulphate bolus (20)	(i) Intraop analgesia requirement (fentanyl)
		(i) Thiopental 5 mg/kg	(ii) Ketamine 0.3 mg/kg (20)	(ii) Time to first analgesia
		(ii) Fentanyl 100 μg at delivery	(iii) 20 mL normal saline control (20)	(iii) Pain (VAS) score

Elrahman and Youssry, 2017, Egypt	90	*General*	(i) IV magnesium sulphate bolus (30)	(i) Total 24 hr analgesia use (morphine)
		(i) IV propofol 2 mg/kg	(ii) Normal saline control (30)	(ii) Time to first analgesia
		(ii) IV midazolam 1 mg and fentanyl 1 μg/kg at delivery		(iii) Pain (VAS) score
				(iv) Time to ambulation

Maulik et al., 2015, India	80	*Spinal*	(i) IV magnesium sulphate bolus and 24 hr infusion (40)	(i) Rescue analgesia consumption (diclofenac)
		(i) Bupivacaine 0.5%	(ii) Normal saline control (40)	(ii) Time to first analgesia
				(iii) Pain (VAS) scores

Mireskandari et al., 2015, Iran	50	*General*	(i) IV magnesium sulphate bolus (25)	(i) Total 24 hr PCA consumption (morphine)
		(i) Thiopental 4 mg/kg	(ii) Normal saline control (25)	(ii) Pain (VAS) score
		(ii) Fentanyl 1 μg/kg and midazolam 1 mg at delivery		

Paech et al., 2006, Australia	120	*Spinal-epidural*	(i) IV magnesium sulphate bolus and 24 hr infusion (42). High dose: 50 mg/kg and 2 g/hr	(i) Total analgesia consumption (meperidine)
		(i) Bupivacaine 0.5%	(ii) IV magnesium sulphate bolus and 24 hr infusion (38). Low dose: 25 mg/kg and 2 g/hr	(ii) Time to first analgesia
		(ii) Fentanyl 15 μg	(iii) Normal saline control (40)	(iii) Pain (VAS) score

Rezae et al., 2014, Iran	70	*General*	(i) Magnesium sulphate 50 mg/kg in 100 mL NS bolus (35)	(i) Total 24 hr analgesia consumption (morphine)
		(i) Thiopental 6 mg/kg		
		(ii) Fentanyl 2 μg/kg	(ii) Normal saline control (35)	(ii) Pain (VAS) score
		(iii) Morphine 0.15 mg/kg after delivery		

	530		*Receiving magnesium* (260)	

IV = intravenous, hr = hours, VAS = visual analogue scale, PCA = patient controlled analgesia.

**Table 3:** Intervention regimens per trial.

	Patient type	Timing of intervention	Dosing protocol
Elgebaly et al., 2011	*Severe preeclampsia* (SBP ≥ 160 mmHg or DBP ≥ 100 mmHg or proteinuria ≥ 100 mg/dL)	(i) Bolus 30 minutes before spinal	(i) *Bolus*: 6 g magnesium sulphate in 100 mL NS over 30 minutes
		(ii) Infusion commenced following bolus	(ii) *Infusion*: 2 g/hr magnesium sulphate for 24 hr
			(iii) *Controls 1 and 2*: 160 mL NS over 30 minutes and then 60 mL NS infusion for 24 hr

Elrahman and Youssry, 2017	*ASA I and II Elective* Excluded preeclampsia patient	(i) Bolus 30 minutes before spinal	(i) *Bolus*: magnesium sulphate 50 mg/kg in 100 mL NS over 20 minutes

Helmy et al., 2015	*ASA I and II Elective* No preeclampsia patients	(i) 10 minutes before induction	(i) *Bolus*: magnesium sulphate 30 mg/kg in 20 mL NS over 10 minutes
			(ii) *Control 1*: ketamine 0.3 mg/kg in 20 mL NS over 10 minutes
			(iii) *Control 2*: 20 mL NS over 10 minutes

Maulik et al., 2015	*Severe preeclampsia* ASA < III BMI 18.5–35	(i) 30 minutes before surgery	(i) *Bolus*: magnesium sulphate 40 mg/kg in 100 mL NS over 15 minutes
		(ii) Infusion commenced following bolus	(ii) *Infusion*: magnesium sulphate 10 mg/kg/hr for 24 hr
			(iii) *Control*: NS of same volume and rate for both bolus and infusion

Mireskandari et al., 2015	*ASA I and II* Hypertension excluded	(i) Before induction	(i) *Bolus*: magnesium sulphate 50 mg/kg in 500 mL NS over 15 minutes
			(ii) *Control*: 500 mL NS over 15 minutes

Paech et al., 2006	*Elective* No preeclampsia patients	(i) One hour before surgery	*Group 1*
			(i) *Bolus*: magnesium sulphate 50 mg/kg
			(ii) *Infusion*: magnesium sulphate 2 g/hr for 24 hr
		(ii) Infusion commenced following bolus	*Group 2*
			(i) *Bolus*: magnesium sulphate 25 mg/kg
			(ii) *Infusion*: magnesium sulphate 1 g/hr for 24 hr
			(iii) *Control*: NS at same volume and rate

Rezae et al., 2014	*Elective*	(i) 30 minutes before induction	(i) *Bolus*: magnesium sulphate 50 mg/kg in 100 mL NS over 10 minutes
			(ii) *Control*: NS at same volume and rate

SBP = systolic blood pressure, DBP = diastolic blood pressure, NS = normal saline, hr = hours, ASA = American Society of Anesthesiologists Physical Status Classification System, BMI = body mass index.

**Table 4:** Postoperative analgesia outcomes.

	Elgebaly et al. (2011)	Elrahman and Youssry (2017)	Helmy et al. (2015)	Maulik et al. (2015)	Mireskandari et al. (2015)	Paech et al. (2006)	Rezae et al. (2014)
Anaesthesia^∗^	S	G + (TAP)	G	S	G	S + (F)	G

Intraoperative opioids^†^	No	Yes (F)	Yes (F)	No	Yes (F)	Yes (F)	Yes (F + M)

Magnesium intervention group (n)	30	30	20	40	25	42, 38	35

Analgesia type^‡^	IV (Pa) PRN	IV (M) PRN	IM (Pe) by VAS	IM (D) by VAS	IV (M) PCA	PO (Pa) and (Me) PCEA	IV (M) PRN

Time to first postoperative analgesia requirement	*Mg* = 7.05 hr	*Mg* = 200 m	*Mg* = 36 m	*Mg* = 270 m	—	*Mg* ^1^ = 86 m	—
	*C* = 3.7 hr	*C* = 120 m	*C* = 33 m	*C* = 223.6 m		*Mg* ^2^ = 102 m	
	(*p* < 0.01)	(*p* < 0.001)	NS	(*p* < 0.001)		*C* = 105 m	
						NS (0.867)	

Total postoperative analgesia consumption (Mg versus C)	*Reported as frequency of analgesia*	*Mg* = 6.2 mg (M)	*Mg* = 137 mg (Pe)	*Mg* = 2.5 g (D)	*Mg* = 4.36 u (M)	*Mg* ^1^ = 565 mg (Me)	*Mg* = 11.2 mg (M)
	*Mg* = 2.5 (0.4)					*Mg* ^2^ = 585 mg (Me)	
	*C* = 3.9 (0.5)	*C* = 10.1 mg (M)	*C* = 140 mg (Pe)	*C* = 3.6 g (D)	*C* = 7.2 u (M)	*C* = 543 mg (Me)	*C* = 13.9 mg (M)

End point	*Freq. (24* *hr)*	24 hr	24 hr	24 hr	24 hr	48 hr	24 hr

*p* value	(*p* < 0.01)	(*p* < 0.01)	NS	(*p* < 0.001)	(*p* < 0.001)	NS (0.792)	(*p* < 0.01)

^∗^S = spinal, G = general, (F) = intrathecal fentanyl, (TAP) = bilateral transversus abdominus plane block, ^†^(F) = fentanyl, (F + M) = fentanyl and morphine, ^‡^IV = intravenous, IM = intramuscular, PO = oral, PRN = as required, VAS = visual analogue scale, PCA = patient-controlled analgesia, PCEA = patient-controlled epidural analgesia, (T) = tramadol, (D) = diclofenac, (Pa) = paracetamol, (Pe) = pethidine, (M) = morphine, (Me) = meperidine, hr = hour, m = minute, g = gram, mg = milligram, u = units not specified, *Mg* = magnesium (*Mg*^1^ = Paech et al. low dose magnesium, *Mg*^2^ = Paech et al. high dose magnesium), *C* = control, NS = not significant.

**Table 5:** Patient-ranked pain scores (VAS): magnesium intervention versus control.

Time point of VAS score assessment	Elgebaly et al. (2011)^∗^	Elrahman and Youssry (2017)^∗^	Helmy et al. (2015)^†^	Maulik et al. (2015)^‡^	Mireskandari et al. (2015)^∗^	Paech et al. (2006)^§^	Rezae et al. (2014)^‡^
Time of first analgesia req.	*Mg* = 34	—	—	—	—	—	—
	*C* = 36						
	NS						

1 hour	—	—	—	—	*Mg* = 48.9	—	—
					*C* = 74.7		
					(*p* < 0.001)		

2 hours	—	*Mg* = 51.3	*Mg* = 3	*Mg* = 1.2	—	—	*Mg* = 3.2
		*C* = 58.7	*C* = 4	*C* = 1.7			*C* = 4.9
		NS	NS	(*p* < 0.001)			(*p* < 0.03)

4 hours	—	—	—	*Mg* = 1.3	—	—	—
				*C* = 1.9			
				(*p* < 0.001)			

6 hours	—	*Mg* = 40.4	*Mg* = 4	—	*Mg* = 42.1	—	—
		*C* = 53.6	*C* = 4		*C* = 58.3		
		(*p* < 0.05)	NS		(*p*=0.002)		

8 hours	—	—	—	*Mg* = 2.7	—	—	—
				*C* = 3.5			
				(*p* < 0.001)			

12 hours	—	*Mg* = 26.1	*Mg* = 3	—	*Mg* = 25.2	—	*Mg* = 2.8
		*C* = 35.5	*C* = 3.5		*C* = 30		*C* = 3.6
		(*p* < 0.01)	NS		(*p*=0.05)		(*p* < 0.03)

16 hours	—	—	—	*Mg* = 1.4	—	—	—
				*C* = 2			
				(*p* < 0.001)			

24 hours	—	*Mg* = 23.3	*Mg* = 2	*Mg* = 0.7	*Mg* = 22.6	—	*Mg* = 1.8
		*C* = 24.2	*C* = 2.5	*C* = 1.3	*C* = 23.6		*C* = 2.9
		NS	NS	(*p* < 0.001)	NS (*p*=0.49)		(*p* < 0.03)

48 hours	—	—	—	—	—	*Mg* ^1^ = 90	—
						*Mg* ^2^ = 83	
						*C* = 90	
						NS (*p*=0.449)	

VAS = visual analogue scale, NS = not significant, ^∗^visual analogue scale (0–100) expressed as mean, ^†^visual analogue scale (0–10) expressed as median, ^‡^visual analogue scale (0–10) expressed as mean, ^§^patient satisfaction visual analogue scale (0–100) expressed as median, *Mg*^1^ = low-dose group, *Mg*^2^ = high-dose group, *Mg* = magnesium intervention group, *C* = control group.
